# Dengue Virus Infection of *Aedes aegypti* Alters Extracellular Vesicle Protein Cargo to Enhance Virus Transmission

**DOI:** 10.3390/ijms21186609

**Published:** 2020-09-10

**Authors:** Alexander S. Gold, Fabiana Feitosa-Suntheimer, Ricardo V. Araujo, Ryan M. Hekman, Sultan Asad, Berlin Londono-Renteria, Andrew Emili, Tonya M. Colpitts

**Affiliations:** 1 Department of Microbiology, National Emerging Infectious Diseases Laboratories, Boston University School of Medicine, 620 Albany Street, Boston, MA 02118, USA; asgold@bu.edu (A.S.G.); araujorv@hotmail.com (R.V.A.); sasad@bu.edu (S.A.); tmcol@bu.edu (T.M.C.); 2 Department of Biochemistry, Boston University School of Medicine, 72 E. Concord Street, Boston, MA 02118, USA; rhekman@bu.edu (R.M.H.); aemili@bu.edu (A.E.); 3 Department of Entomology, Vector Biology Laboratory, Kansas State University, 1603 Old Claflin Pl, 123 Waters Hall, Manhattan, KS 66506, USA; blondono@ksu.edu

**Keywords:** dengue virus, *Aedes aegypti*, extracellular vesicles

## Abstract

Dengue is the most burdensome vector-borne viral disease in the world. Dengue virus (DENV), the etiological cause of dengue, is transmitted primarily by the *Aedes aegypti* mosquito. Like any arbovirus, the transmission cycle of dengue involves the complex interactions of a multitude of human and mosquito factors. One point during this transmission cycle that is rich in these interactions is the biting event by the mosquito, upon which its saliva is injected into the host. A number of components in mosquito saliva have been shown to play a pivotal role in the transmission of dengue, however one such component that is not as well characterized is extracellular vesicles. Here, using high-performance liquid chromatography in tandem with mass spectrometry, we show that dengue infection altered the protein cargo of *Aedes aegypti* extracellular vesicles, resulting in the packaging of proteins with infection-enhancing ability. Our results support the presence of an infection-dependent pro-viral protein packaging strategy that uses the differential packaging of pro-viral proteins in extracellular vesicles of *Ae. aegypti* saliva to promote transmission. These studies represent the first investigation into the function of *Ae. aegypti* extracellular vesicle cargo during dengue infection.

## 1. Introduction

Dengue virus (DENV) is a mosquito-borne virus responsible for a greater burden of human disease than any other arbovirus, causing an estimated 10,000 deaths and 100 million symptomatic infections per year in over 125 countries [1,2,3]. Dengue is a flavivirus most commonly transmitted by *Aedes aegypti*, which flourish in tropical and sub-tropical urban settings around the world [4]. As a result, about one half of the global population lives in areas environmentally suitable for DENV transmission [5,6]. Recently, due to the increased epidemic activity and geographic expansion of DENV infection and *Ae. aegypti*, respectively, DENV is now considered an emerging global health threat [7,8,9,10]. Due to climate change and population growth, models predict that 2.25 billion more people will be at risk for dengue in 2080 compared to 2015, bringing the total population at risk to over 6.1 billion [11]. Although a dengue vaccine is now available, its long-term protective efficacy against all four serotypes has not been established, and consequently novel mechanisms for prophylactics and therapeutics for dengue infection are still urgently needed [12]. Identifying molecules involved in the transmission of DENV to the vertebrate host is a crucial first step in the development of new drugs and vaccines [13].

One component of the DENV transmission cycle that offers plenty of potential targets is mosquito saliva, which is injected into human skin along with virus during an infectious bite and has been shown to alter virus infection in mammals [14,15,16]. Mosquito saliva contains a mixture of proteins that function collectively to allow the mosquito to acquire a blood meal by overcoming host barriers such as vasoconstriction, platelet aggregation, coagulation, and inflammation [17,18,19]. Many proteins found in mosquito saliva are known to be immunogenic, often resulting in allergic reactions ranging in severity and indicating the capacity of proteins in saliva to influence viral infection [20,21]. Previous studies examining the contents of *Ae. aegypti* saliva have shown that it contains over one hundred unique proteins, many of which have been shown to enhance or inhibit DENV infection [18,22,23]. In order for these salivary proteins to potentially affect DENV infection in a mammalian host, they must either be secreted directly into the saliva or packaged in extracellular vesicles, which are then secreted into the saliva.

Extracellular vesicles (EVs), including microvesicles and exosomes, are believed to be secreted by all eukaryotic cells [24,25,26]. Made up of a highly heterogenous population of membrane-bound vesicles of differing origin and cargo, EVs are considered a mechanism of intercellular communication, and as a result are involved in a multitude of physiological and pathological functions [25,26]. Only lately was it demonstrated that a cell line from a medically important arthropod secreted EVs [27,28,29]. While the role of mosquito EVs in viral infection remains unclear, several recent studies have shown that arthropod EVs are capable of mediating flavivirus transmission and that flavivirus infection affects EV cargo [27,28,30].

In this study, we examined how the protein cargo of *Ae. aegypti* EVs differed upon DENV infection and how changes in the cargo impacted infection. We compared and cross-referenced this data with saliva proteins that we found were able to enhance DENV infection in mammalian cells. By measuring the DENV infection-enhancing ability of proteins found in *Ae. aegypti* saliva and determining what proteins were also present in *Ae. aegypti* EVs, we identified a DENV infection-enhancing protein that was selectively packaged in mosquito EVs during DENV infection. The identification of infection-enhancing molecules found in arthropod EVs points to novel transmission mechanisms, many of which could allow for the development of therapeutics and insect-based transmission vaccines against identified targets that would impede transmission and infection in humans.

## 2. Results

### 2.1. Identification of DENV Infection-Enhancing Aedes aegypti Saliva Proteins

**Figure 1:**
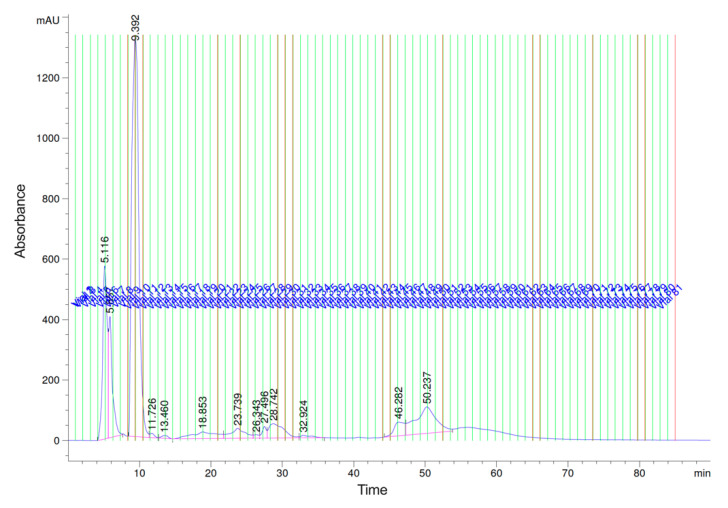
Saliva was collected from female *Ae. aegypti* after blood feeding. Mosquito saliva was fractionated by high-performance liquid chromatography (HPLC) on a nonporous reverse-phase column with a TFA buffer system into 80 fractions of 100 μL each.

Mosquito saliva as a whole has been shown to enhance infection in mammals for several arboviruses, yet there is a lack of information regarding the identification of individual salivary proteins with an impact on viral infection. Our group and others have identified several proteins in *Ae. aegypti* saliva that have either enhancing or inhibitory effects on DENV infection in mammalian cells [18,22]. To measure the effect of individual salivary proteins on DENV infection, we used a method developed and optimized in our lab to separate (fractionate) saliva proteins, evaluate the impact of each fraction on DENV infection in human cells, and then identify the proteins present in fractions with infection-enhancing properties. Unlike previous studies, which isolated proteins from homogenized salivary gland tissues (termed salivary gland extract or SGE), here we used actual mosquito saliva to ensure that the identified proteins have relevance in mosquito–human transmission in nature. To do this, saliva was collected from blood-fed *Ae. aegypti* and fractionated by high-performance liquid chromatography (HPLC), which separated proteins into 80 fractions on the basis of their hydrophobicity (Figure 1) [18,22].

**Figure 2:**
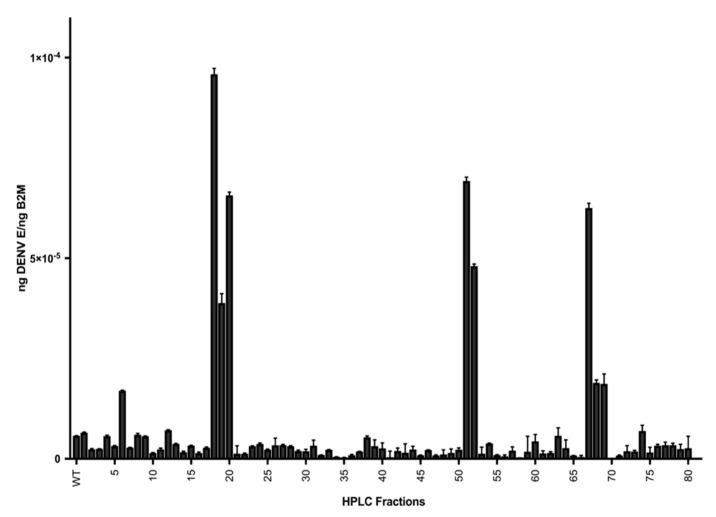
Human primary dermal fibroblast cells were simultaneously treated with 80 *Aedes aegypti* saliva fractions and infected with DENV-2. Wild-type (WT) cells were treated with whole saliva and DENV-2, instead of saliva fractions. Then, 24 h post-infection, the cells were lysed and RNA was collected and used to quantify the DENV viral load by qRT-PCR. Data presented are ng RNA, calculated using previously determined standard curves for DENV E mRNA and Human B2M mRNA. Results are the mean ± standard error of the mean of three independent experiments.

Following fractionation, to assess the effect of salivary proteins on DENV infection, human fibroblasts were simultaneously treated with saliva fractions and infected with DENV. RNA was isolated from these fibroblasts 24 hours post-infection and used to quantify the DENV infection by qRT-PCR. When compared to control cells infected with DENV but untreated with any saliva fractions, many saliva fractions had an impact on infection, with several demonstrating substantial infection-enhancing activity (Figure 2). To identify which proteins were present and putatively responsible for greater levels of infection in the fibroblasts, aliquots of the saliva fractions were subjected to LC/MS-MS analysis, and these data were used to generate a list of proteins found in each fraction (Table 1).

### 2.2. DENV Infection Altered Protein Cargo of Ae. aegypti EVs

**Figure 3:**
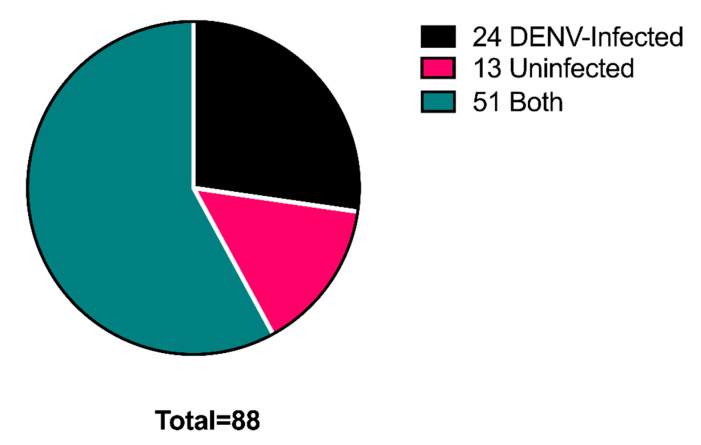
Extracellular vesicles were isolated from DENV-infected and uninfected *Ae. aegypti* cells. Proteins from both EV samples were processed and identified by mass spectrometry. Of the 88 total proteins identified, while the majority were observed in the EVs from both infected and uninfected cells (51, 58%), several were observed only in the EVs derived from DENV-infected cells (24, 27%) and in EVs from uninfected cells (13, 15%).

Although the ability of mosquito EVs to mediate flavivirus infection in vitro has been reported, the protein cargoes of these EVs remained to be determined [27]. In addition, it is unknown whether differentially packaged proteins could impact infection via the EVs. To assess how DENV infection affected extracellular vesicular protein packaging, EVs were isolated from DENV-infected and uninfected *Ae. aegypti* cells. The total protein was isolated from these EVs, and samples were subjected to LC/MS-MS to determine which proteins were found in the EVs from DENV-infected cells, uninfected cells, or both EV sample types (infected and uninfected). The majority of the proteins identified were found in both uninfected and infected cells, which was expected as extracellular vesicles contain a wide range of proteins that are necessary for their functions (Figure 3). However, several proteins were exclusively found in the EVs derived from DENV-infected cells (Figure 3 and Table 2), supporting an infection-dependent protein packaging strategy, similar to that previously observed in DENV-infected human dendritic cells [31].

### 2.3. DENV Infection Resulted in the Packaging of Infection-Enhancing Cargo in Ae. aegypti EVs

**Figure 4:**
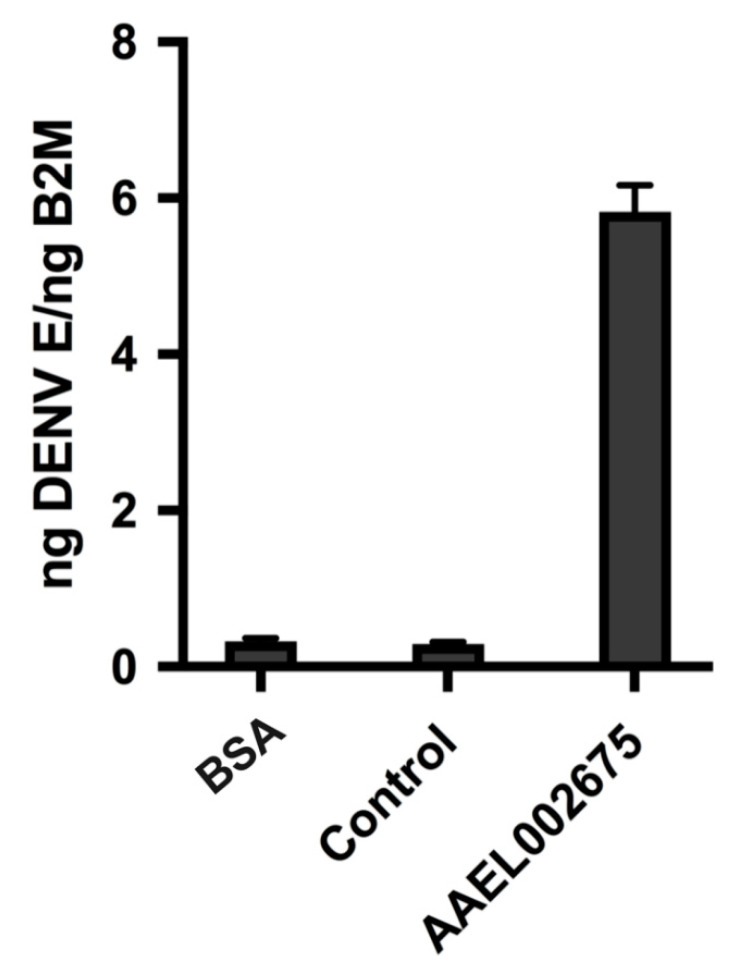
Human primary dermal fibroblast cells were treated with either BSA, control (AAEL001928; Actin-1), or AAEL002675 for one hour before infection with DENV-2 at a MOI of 0.1. Then, 24 h post-infection, the cells were lysed and RNA was collected and used to quantify the DENV viral load by qRT-PCR. Data presented are ng RNA calculated using previously determined standard curves for DENV E mRNA and Human B2M mRNA. Results are the mean ± standard error of the mean of three independent experiments.

Twenty-four proteins were identified only in EVs isolated from DENV-infected *Aedes aegypti* cells (Table 2). Putative functions were identified for several of these proteins, and included receptors, proteases, and cuticle proteins, among others. Of these proteins, AAEL002675 was the only protein found in EVs derived from DENV-infected cells and in multiple fractions of mosquito saliva that displayed infection-enhancing activity in vitro (Figure 2). To demonstrate the intrinsic DENV infection-enhancing ability of AAEL002675, the recombinant protein was overexpressed in *Drosophila melanogaster* cells, purified, and used to pre-treat human fibroblast cells before infection with DENV. The addition of AAEL002675 to these cells resulted in the robust enhancement of the DENV viral load (Figure 4), greatly supporting the infection-enhancing ability of this protein.

### 2.4. AAEL002675 Expression Increased upon DENV Infection

**Figure 5:**
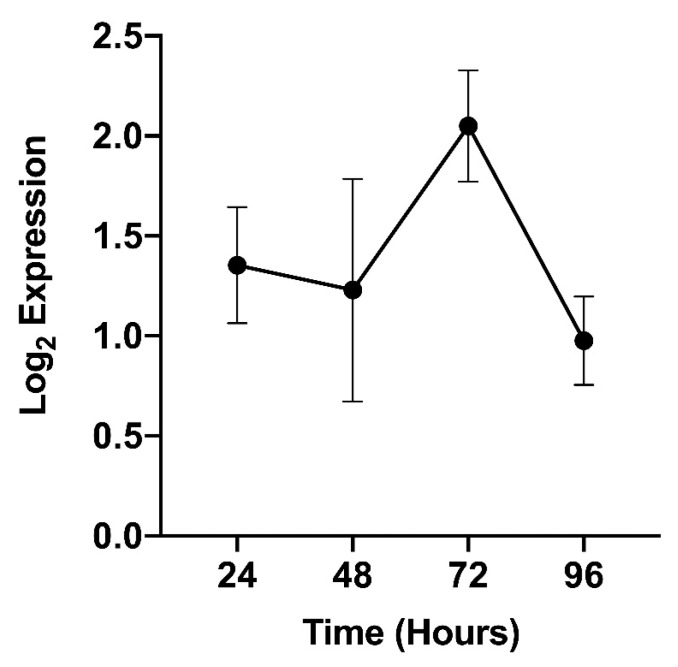
*Ae. aegypti* cells were infected with DENV-2. At the given timepoints after infection, the cells were lysed and RNA was collected and used to quantify the AAEL002675 gene expression by qRT-PCR. Results are the mean ± standard error of the mean of three independent experiments.

To better understand the DENV infection-dependent packaging of AAEL002675 in EVs, it was important to examine how DENV infection affected the expression of AAEL002675 in *Ae. aegypti*. In doing so, *Ae. aegypti* cells were infected with DENV and the level of AAEL002675 gene expression was measured from 24 to 96 hours post-infection (Figure 5). At all times following infection, an increase in the expression of AAEL002675 was observed, with a pronounced peak of expression at 72 hours post-infection.

## 3. Discussion

Proteins found in *Ae. aegypti* saliva have been shown to enhance DENV replication and spread [18,23,32]. By using our unique method of saliva protein analysis, we identified multiple proteins with suspected DENV infection-enhancing ability (Table 1). Additionally, while the ability of *Aedes* EVs, a widely-believed component of mosquito saliva, to facilitate DENV transmission has been demonstrated, the functions of the protein cargo of these EVs during infection remain to be determined [27]. Our data (Figure 3 and Table 2) show that DENV infection impacts the cargo packaging of *Ae. aegypti* EVs, a phenomenon previously characterized using EVs derived from DENV-infected dendritic cells [31]. Several of the proteins found in EVs derived from infected cells, but not uninfected cells, have known infection-enhancing putative functions (Table 2). These results support the presence of a DENV infection-dependent protein packaging strategy, aimed at increasing viral transmission through the delivery of infection-enhancing cargo by vesicular trafficking. Such a pro-viral strategy has been described for a multitude of other clinically relevant viruses, including HIV, Epstein–Barr virus, Cytomegalovirus, and Hepatitis C virus, however this is the first report of this strategy as a mechanism of DENV transmission [26].

Of the proteins found only in EVs from infected cells, two with well-characterized functions during DENV infection are AAEL017301, the *Ae. aegypti* elongation factor-1 alpha (EF1A); and AAEL017982, the *Ae. aegypti* heat shock protein 70 (HSP70) (Table 2). Human EF1A has been shown to function in the replication and pathogenesis of a diverse group of RNA viruses [33]. In the case of West Nile virus, a mosquito-borne member of the *Flavivirus* genus, human EF1A has been shown to facilitate viral minus-strand RNA synthesis through interaction with the 3′-terminal stem-loop region of the viral genome [34,35]. Similarly, the EF1A of *Ae. albopictus* has been shown to bind to the 3′ untranslated region of DENV in cells [36]. Although neither of these studies support the function of *Ae. aegypti* EF1A in DENV infection, the expression of AAEL017301 has been shown to increase following DENV infection, indicating a pro-viral role similar to that demonstrated in previous work [37]. Like EF1A, many studies have shown the involvement of HSP70 in flavivirus infection [38,39]. A HSP70 chaperone network has been shown to mediate the DENV viral cycle, in that cytosolic HSP70 isoforms are required at distinct steps, including entry, RNA replication, and virus assembly [38,39]. A similar HSP70 chaperone network has been described for Zika virus, another mosquito-borne flavivirus similar to DENV [40]. Importantly, the inhibition of HSP70 blocks DENV replication as well as the replication of other mosquito-borne flaviviruses, supporting the role of *Ae. aegypti* HSP70 in the context of viral transmission and spread [38]. Similar to both of these proteins, another protein found EVs derived from infected cells, AAEL002675, has a function known to affect viral infection.

AAEL002675, an *Ae. aegypti* arginase, was the only protein identified in both the DENV infection-enhancing saliva fractions (Table 1) and EVs derived from DENV-infected *Aedes* cells (Table 2) [41]. Although studies have elucidated the physiological role of *Ae. aegypti* arginase, the function of this protein during DENV infection remains undefined [42,43]. However, much more work has been performed characterizing the function of human arginase I (Arg-I), the human homolog of AAEL002675. Physiologically, Arg-I metabolizes L-arginine to L-ornithine and urea, serving a fundamental role in the hepatic urea cycle [44]. Yet, several types of immune cells require L-arginine for their effector functions [44]. For example, activated macrophages consume L-arginine by converting it to L-citrulline, nitric oxide (NO), and reactive nitrogen species via induced nitric oxide synthase as their primary mechanism of cytostatic or cytotoxic activity against viruses [45]. Due to this role in the innate immune response, Arg-I has been shown to play a role in several viral infections [46]. For example, the expression of Arg-I has been shown to be associated with an increased viral load, disease severity, and persistence of the Chikungunya virus, which, like DENV, is transmitted by the *Ae. aegypti* mosquito [47].

Although the role of Arg-I during DENV infection is not well known, it has been shown to promote the persistence of the flavivirus Hepatitis C virus in human hepatoma cells [48]. Additionally, the function of NO during DENV infection has been characterized, and while the exact mechanism has yet to be determined, it has been shown to inhibit DENV replication by impeding the polymerase activity of the viral protein NS5 [49,50]. In this case, if an exogenous arginase, such as AAEL002675, was delivered to recently infected cells, cytosolic L-arginine would be converted to L-ornithine and urea, thereby reducing the cytosolic concentration of L-arginine that could be converted to NO and subverting the desired immune response. Our data show that AAEL002675 enhances DENV infection in human fibroblasts (Figure 4), strongly supporting the ability of this protein to disrupt the anti-viral immune response, perhaps similarly to Arg-I by presumably converting cytosolic L-arginine and limiting the ability of NO to be produced. Additionally, not only does AAEL002675 enhance DENV infection, but the DENV infection of *Ae. aegypti* results in an increase in the expression of AAEL002675 (Figure 5), which would only result in a greater conversion of cytosolic L-arginine and a lesser amount of cellular NO.

The incorporation of infection-enhancing proteins such as AAEL002675, AAEL017301, and AAEL017982 in EVs derived from DENV-infected *Ae. aegypti* cells suggests the presence of a pro-viral vesicular protein packaging strategy upon DENV infection. While the ability of *Aedes* EVs to mediate DENV transmission has been characterized, this is the first report demonstrating the packaging of the infection-enhancing cargo in the EVs of *Aedes*, representing a novel mechanism of DENV infection and transmission [27]. Previous studies have investigated the functions of secreted proteins in saliva on DENV transmission, however our results show that considering the role of salivary EV protein cargo in viral transmission and spread is a topic of equal importance. Identifying more molecules involved in the transmission of DENV such as those described here is a positive step forward in the development of antiviral therapeutics and vaccines.

## 4. Materials and Methods

### 4.1. Mosquito Rearing and Saliva Collection

*Aedes aegypti* Rockefeller strain were kept in insectary conditions at 28 °C, 80% humidity, and light:dark cycle (12 h:12 h). Saliva from 8 to 10-day-old female mosquitoes was collected following the protocols described elsewhere with small modifications [51,52]. Briefly, a plexiglass (25.5 cm × 17.5 cm) was used as platform to place the mosquitoes. Sterile, 200 μL pipette tips were then filled with 50 μL of sterile 1× PBS and secured on the plexiglass using a clear tape to collect saliva. Wings and legs were removed from cold anesthetized mosquitoes, and the proboscis was inserted into the pipette tip. Mosquitoes were allowed to salivate for 30 min, then pipette tip was removed, and the content was pooled (200 mosquitoes/pool) in a sterile 1.5 mL tube. Saliva pools were kept at −80 °C until use. The protein concentration of the saliva was verified using the NanoDrop 1000 spectrophotometer (Thermo Fisher Scientific, Waltham, MA, USA).

### 4.2. Determination of Enhancing Salivary Proteins by HPLC and LC-MS/MS

Mosquito saliva was fractionated by high-performance liquid chromatography (HPLC) on a nonporous reverse-phase column with a trifluoroacetic acid (TFA) buffer system into 80,100 μL fractions. Primary Dermal Fibroblast; Normal, Human, Adult (HDFa) (ATCC^®^ PCS-201-012™, Manassas, VA, USA) were cultured with DMEM (Gibco™ 11995065, Thermo Scientific, Waltham, MA, USA) containing 10% FBS (Gemini 100–106) and 1% penicillin/streptomycin (Gibco™ 15140163, Thermo Scientific, Waltham, MA, USA) at 37 °C with 5% CO_2_. Saliva fractions (final protein concentration 1 μg/mL) were incubated with Dengue Virus type 2, New Guinea Strain (DENV-2-NGC, MOI of 0.1) for 15 min at 37 °C. After this time, the saliva fraction/virus mixture was added to the cells. The wild-type (WT) group for this experiment were fibroblasts treated with a saliva/virus mixture prepared the same way as those used to treat the experimental groups except using whole, unfractionated saliva. This experiment was performed in three biological replicates (*n* = 3). Fibroblasts were lysed 24 h post-infection, and RNA was isolated using the RNeasy Plus Mini Kit (Qiagen™ 74136, Hilden, Germany) according to the manufacturer’s instructions. To determine the infection-modifying effect of each saliva fraction, this RNA was used to measure the DENV-2 viral load by quantifying the amount of DENV-2 viral RNA normalized to human β2 microglobulin (B2M) RNA by qRT-PCR using the QuantiFast SYBR Green PCR Kit (Qiagen™ 204056, Hilden, Germany) according to the manufacturer’s instructions. qRT-PCR was performed in duplicate using an RNA volume of 2.5 μL (40 ng). The primers used for the qRT-PCR reactions to quantify DENV-2 RNA were designed to target the region of DENV-2 genome that encoded for the virus’ envelope protein (E) based on the sequence of DENV-2 (NC_001474) [53]. The primers used for the qRT-PCR reactions to quantify human B2M were designed using the known human B2M gene sequence (NC_000015.10) [54]. qRT-PCR was performed using a Bio-Rad C1000™ thermal cycler (Hercules, CA, USA) combined with a Bio-Rad CFX96^™^ (Hercules, CA, USA) detection module. The primers used include: DENV E_F: CATTCCAAGTGAGAATCTCTTTGTCA, DENV E_R: CAGATCTCTGATGAATAACCAACG; Human B2M_F: CTCCGTGGCCTTAGCTGTG, Human B2M_R: TTTGGAGTACGCTGGATAGCC. The fractions that enhanced DENV infection were submitted for liquid chromatography tandem mass spectrometry (LC+MS/MS) analysis. This work was performed by the Interdisciplinary Center for Biotechnology Research at the University of Florida in Gainesville, FL. An *Ae. aegypti* database was used for searching using Mascot-generated files from the MS/MS spectra.

### 4.3. Aedes aegypti Extracellular Vesicle Isolation and Protein Processing

*Aedes aegypti* (ATC-10) (ATCC^®^ CCL-125™, Manassas, VA, USA) cells were cultured in DMEM (Gibco™ 11995065) containing 10% FBS (Gemini 100–106), 1% penicillin/streptomycin (Gibco™ 15140163), and 1% tryptose phosphate broth (Gibco™ 18050039) at 30 °C with 5% CO_2_. The cells were treated with Dengue Virus type 2, New Guinea Strain (DENV-2-NGC) at MOI of 1.0. Following 2 h of treatment with DENV-2, the media and virus were aspirated, and the cells were washed three times with sterile PBS (Gibco™ 10010049) and cultured in DMEM containing 10% exosome-depleted FBS (Gibco™ A2720803, Thermo Scientific, Waltham, MA, USA), 1% penicillin/streptomycin (Gibco™ 15140163), and 1% tryptose phosphate broth (Gibco™ 18050039, Thermo Scientific, Waltham, MA, USA) for 72 h. The cell supernatants following infection or not were collected and used to isolate extracellular vesicles using Invitrogen™ Total Exosome Isolation Reagent (Invitrogen™ 4478359, Thermo Scientific, Waltham, MA, USA), a method previously described for the isolation of EVs from *Aedes* cells [27,55], according to the manufacturer’s instructions. The proteins contained in these EVs were processed using the Thermo Scientific Pierce In-Solution Tryptic Digestion and Guanidination Kit (Thermo Scientific™ 89895, Waltham, MA, USA) according to the manufacturer’s instructions.

### 4.4. Mass Spectrometry

Each sample of digested peptides was desalted with a ZipTip^®^ with 0.6 μL resin bed volume (Millipore, Burlington, MA, USA) according to the manufacturer’s instructions. The eluent was dried and resuspended in 0.1% formic acid, and was analyzed using a Q Exactive HFX mass spectrometer connected to an Easy nLC 1200 ultra high-pressure chromatography system (Thermo Scientific, Waltham, MA, USA). The samples were loaded onto a reverse-phase nano-trap column (75 μm interior diameter × 2 cm, Acclaim PepMap100 C18 3 μm, 100 Å, Thermo Fisher Scientific, Waltham, MA, USA) with mobile phase A (0.1% formic acid and 2% acetonitrile), and separated over an EASY-Spray column, (ES803A, 75 μm i.d. × 50 cm C18 2 μm, 100 Å, Thermo Fisher Scientific, Waltham, MA, USA) using a gradient (2% to 32% over 60 min) of mobile phase B (0.1% formic acid, 80% acetonitrile) at a flow rate of 250 nL/min. The mass spectrometer was operated in positive ion mode with a capillary temperature of 275 °C and a potential of 2100 V applied to the emitter. All the data were acquired with the mass spectrometer operating in automatic data dependent switching mode. A high-resolution (60,000) MS precursor ion scan (350–1500 *m*/*z* range) was performed to select the 10 most intense ions for the subsequent fragmentation and MS/MS analysis using HCD (NCE 29 at 15,000 resolution) each duty cycle.

### 4.5. Peptide Identification

The resulting RAW files were individually converted and searched using the MaxQuant platform (version 1.6.0.16; http://maxquant.org/) under standard settings. Searches were performed twice, once against all Uniprot entries in both Swiss-Prot and TrEMBL for *Aedes aegypti* (Taxonomy ID: 7159, downloaded 12 December 2018) and again against the vectorbase database for *Aedes aegypti* (downloaded 31 January 2019). Searches allowed for two missed trypsin cleavage sites, and variable modifications of N-terminal acetylation and methionine oxidation. The carbamidomethylation of cysteine residues and guanidination of lysine residues were set as a fixed modification.

### 4.6. Cloning of Recombinant Mosquito Protein AAEL002675

RNA from *Aedes aegypti* was isolated using the RNeasy Plus Mini Kit (Qiagen™ 74136, Hilden, Germany) according to manufacturer’s instructions. A total of 5 μg of this RNA was then used to synthesize cDNA using the SuperScript III First-Strand Synthesis Super Mix (Thermo Fisher Scientific 18080400, Waltham, MA, USA). The gene encoding for AAEL002675 was amplified using this cDNA using the Phusion^®^ High-Fidelity PCR Master Mix with HF Buffer (New England BioLabs^®^ M0531S, Ipswich, MA, USA) with primers designed for cloning of the PCR amplicon into the BglII and ApaI cut sites of the pMT/BiP/V5-His *Drosophila* expression vector (Invitrogen™ V413020). The PCR product of this reaction was cleaned using the QiaQuick PCR and Gel Cleanup Kit (Qiagen™ 28506, Hilden, Germany) according to the manufacturer’s instructions. Both the PCR amplicon encoding for the mosquito protein of interest and the pMT/BiP/V5-His expression vector were digested using FastDigest BglII (Thermo Scientific™ FD0083, Waltham, MA, USA) and FastDigest ApaI (Thermo Scientific™ FD1414, Waltham, MA, USA) according to the manufacturer’s instructions. The digested DNA was run on a 1% agarose gel then purified using the QiaQuick PCR and Gel Cleanup Kit (Qiagen™ 28506, Hilden, Germany) according to the manufacturer’s instructions. The digested PCR amplicon encoding for the mosquito protein of interest was ligated with the digested pMT/BiP/V5-His expression vector using T4 DNA Ligase (New England BioLabs^®^ M0202S, Ipswich, MA, USA), according to the manufacturer’s instructions. The product of this ligation reaction was used to transform One Shot™ MAX Efficiency™ DH5α-T1^R^ Competent Cells (Invitrogen™ 12297016, Thermo Scientific, Waltham, MA, USA) according to the manufacturer’s instructions. The cloned plasmid was isolated from clones generated from the transformation reaction using the ZymoPURE™ Plasmid Miniprep Kit (Zymo Research D4212, Irvine, CA, USA). The successful cloning of the gene encoding the mosquito protein of interest in the pMT/BiP/V5-His *Drosophila* expression vector was then confirmed by the sequencing of the cloned plasmid.

### 4.7. Expression, Purification, and Western Blotting of Mosquito Proteins

**Figure A1:**
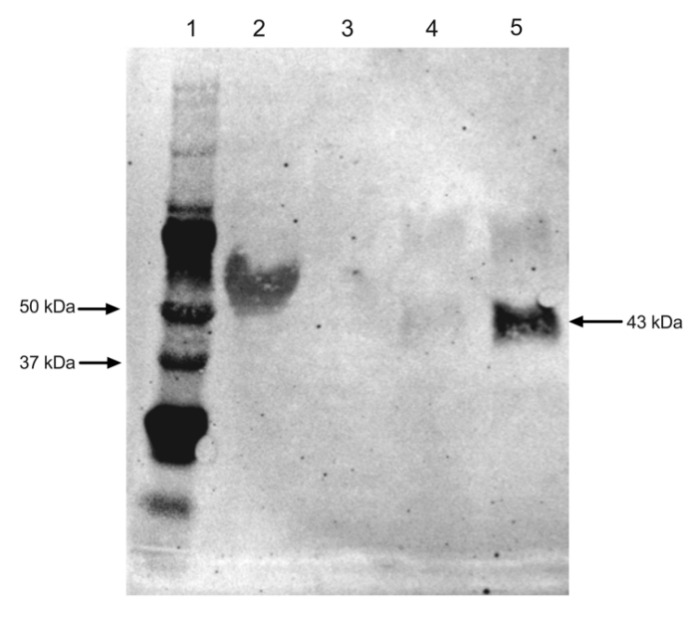
Following purification of the recombinant AAEL002675 protein. Proteins were resolved via SDS-PAGE and probed for the presence of a V5 tag on the protein of interest. Recombinant AAEL002675 (43 kDa) was detected in the first elution fraction from the protein purification process. Lane 1: Precision Plus Protein Dual Color Standards™ (Bio-Rad 1610374); lane 2: S2 supernatant HisPur column flow-through; lane 3: elution fraction 3; lane 4: elution fraction 2; lane 5: elution fraction 1.

Schneider’s Drosophila Line 2 (S2) cells [D. Mel. (2), SL2] (ATCC^®^ CRL-1963™, Manassas, VA, USA) were cultured in Schneider’s Drosophila Medium (Gibco™ 21720024) containing 10% FBS (Gemini 100–106) and 1% penicillin/streptomycin (Gibco™ 15140163) at 28 °C. S2 cells were seeded in T75 flasks and grown to about 80% confluency at 28 °C. Each flask of these cells was then transfected with 10 μg of the previously cloned plasmid containing the gene encoding for the mosquito protein of interest using the Effectene Transfection Reagent (Qiagen™ 301425, Hilden, Germany) according to the manufacturer’s instructions, with the addition of CuSO_4_ to a final concentration of 500 μM to induce protein expression. The cell supernatant was collected 72 h post-transfection, and the recombinant mosquito protein of interest was purified using a HisPur™ Cobalt Spin Column (Thermo Scientific™ 89969, Waltham, MA, USA). The proteins were resolved via SDS-PAGE using a 4–20% Mini-PROTEAN^®^ TGX™ precast protein gel (Bio-Rad 4651094, Hercules, CA, USA). Once resolved, the proteins were transferred to a nitrocellulose membrane using the Trans-Blot^®^ Turbo™ transfer system (Bio-Rad 17001918, Hercules, CA, USA). The nitrocellulose membrane was incubated in blocking 5% skim milk in TBS with 0.05% Tween 20 (blocking buffer) overnight at 4 °C, then washed three times with TBS containing 0.05% Tween 20 (washing buffer), and incubated with V5 Tag Monoclonal Antibody (Thermo Fisher Scientific R960–25, Waltham, MA, USA) overnight at 4 °C, according to the manufacturer’s instructions. The nitrocellulose membrane was then washed three times and incubated with IRDye^®^ 800CW Goat anti-Mouse IgG Secondary Antibody (Li-Cor^®^ Biosciences 925-32210, Lincoln, NE, USA) for two hours at room temperature, according to the manufacturer’s instructions. The protein signal was detected using the Odyssey^®^ CLX300 Near-Infrared Fluorescence Imaging System (Lincoln, NE, USA) (Figure A1 in Appendix A). Once the purity was confirmed, the protein concentration was measured by the Micro BCA™ Protein Assay Kit (Thermo Scientific™ 23235, Waltham, MA, USA) according to the manufacturer’s instructions.

### 4.8. Treatment of Fibroblasts with Mosquito Proteins and Infection with DENV-2

Primary Dermal Fibroblast; Normal, Human, Adult (HDFa) (ATCC^®^ PCS-201-012^™^, Manassas, VA, USA) were cultured with DMEM (Gibco™ 11995065) containing 10% FBS (Gemini 100–106) and 1% penicillin/streptomycin (Gibco™ 15140163) at 37 °C with 5% CO_2_. The cells were treated with proteins at a final concentration of 1 ng/mL for one hour and then infected with Dengue Virus type 2, New Guinea Strain (DENV-2-NGC), at a MOI of 0.1 and kept in the same conditions until use. In this case, the control group consisted of cells were treated with AAEL001928 (*Ae. aegypti* Actin-1), which was previously observed in *Ae. aegypti* salivary gland extract and showed no enhancement of inhibition of DENV infection [22]. The AAEL001928 protein was cloned, synthesized, and purified using the same methods used to produce the AAEL002675 protein. The BSA group consisted of cells treated with bovine serum albumin instead of mosquito protein. This experiment was performed in three biological replicates (*n* = 3). Then, 24 h post-infection, the fibroblasts were lysed and the RNA was isolated using the RNeasy Plus Mini Kit (Qiagen™ 74136, Hilden, Germany) according to the manufacturer’s instructions. The total RNA was used to measure the DENV-2 viral load by quantifying the amount of DENV-2 viral RNA normalized to human β2 microglobulin (B2M) RNA by qRT-PCR using the QuantiFast SYBR Green PCR Kit (Qiagen™ 204056, Hilden, Germany) according to the manufacturer’s instructions. qRT-PCR was performed in duplicate using an RNA volume of 2.5 μL (40 ng). The primers used for the qRT-PCR reactions to quantify DENV-2 RNA were designed to target the region of DENV-2 genome that encoded for the virus’ envelope protein (E) based on the sequence of DENV-2 (NC_001474) [53]. The primers used for the qRT-PCR reactions to quantify human B2M were designed using the known human B2M gene sequence (NC_000015.10) [54]. qRT-PCR was performed using a Bio-Rad C1000™ thermal cycler (Hercules, CA, USA) combined with a Bio-Rad CFX96^™^ detection module (Hercules, CA, USA). The primers used include: DENV_E_F: CATTCCAAGTGAGAATCTCTTTGTCA, DENV_E_R: CAGATCTCTGATGAATAACCAACG; Human B2M_F: CTCCGTGGCCTTAGCTGTG, Human B2M_R: TTTGGAGTACGCTGGATAG CC.

### 4.9. AAEL002675 Expression Changes upon DENV Infection

*Aedes aegypti* (ATC-10) (ATCC^®^ CCL-125™, Manassas, VA, USA) cells were cultured with DMEM (Gibco™ 11995065) containing 10% FBS (Gemini 100–106), 1% penicillin/streptomycin (Gibco™ 15140163), and 1% tryptose phosphate broth (Gibco™ 18050039) at 30 °C with 5% CO_2_. The cells were infected with Dengue Virus type 2, New Guinea Strain (DENV-2-NGC), at a MOI of 0.1 and kept in the same conditions until use. This experiment was performed in three biological replicates (*n* = 3). The cells were lysed 24 h post-infection and the RNA was isolated using an RNeasy Plus Mini Kit (Qiagen™ 74136, Hilden, Germany) according to the manufacturer’s instructions. RNA was used to measure the AAEL002675 expression levels by qRT-PCR using the QuantiFast SYBR Green PCR Kit (Qiagen™ 204056, Hilden, Germany) according to the manufacturer’s instructions. qRT-PCR was performed using a Bio-Rad C1000™ thermal cycler (Hercules, CA, USA) combined with a Bio-Rad CFX96™ detection module (Hercules, CA, USA). qRT-PCR was performed in duplicate using an RNA volume of 2.5 μL (40 ng). The primers used for the qRT-PCR reactions to quantify AAEL002675 RNA were designed based on the mRNA sequence of AAEL002675 (XM_001662007) [41]. The primers used for the qRT-PCR reactions to quantify the *Aedes aegypti* actin RNA were designed based on the mRNA sequence of *Aedes aegypti* actin (DQ440059) [56]. The expression levels were normalized to *Aedes aegypti* actin as the housekeeping gene. Log_2_ expression was calculated using the normalized expression values of AAEL002675 calculated from the measured C_q_ values of AAEL002675 and *Aedes aegypti* actin from the RNA of infected and uninfected cells. The primers used include.: AAEL002675_F: CGGTATCCACGCTTTTGGGA, AAEL002675_R: GGAGCCTCAAGGACATCCAG; *Aedes aegypti* actin_F: GAACACCCAGTCCTGACA, *Aedes aegypti* actin_R: TGCGTCATCTTCTCACGGTTAG.

## Figures and Tables

**Table 1:** Proteins found in the enhancing fractions of mosquito saliva.

Protein SeqID	Fraction Observed	Putative Function-Vector Base ^1^	Putative Function-Blastp ^2^	Accession
AAEL000299-PA	A20	Unknown	Zinc finger protein 490 (*Aedes aegypti*)	XP_001654769.2
AAEL000794-PA	A18, A19, A20, A51, A52	Unknown	Clustered mitochondria protein homolog	Q17N71.1
AAEL000913-PA	A19, A20, A68	Cuticle Protein	Cuticle Protein	XP_001651656.2
AAEL002675-PA	A18, A19, A20, A52	Arginase	Arginase	XP_001662057.1
AAEL005493-PA	A18, A19, A20, A52, A69	Septin	Septin-1 isoform X2	XP_021704188.1
AAEL006525-PA	A20, A52	Kelch Repeat Protein	Kelch domain-containing protein 3	XP_001652008.2
AAEL006528-PA	A20	No match	AAEL006528-PA (*Aedes aegypti*)	EAT41874.1
AAEL006844-PA	A19, A20, A52	GPCR Octopamine/Tyramine Family	Probable G-protein coupled receptor No18	XP_001652255.3
AAEL007354-PA	A67	Pseudouridylate synthase	tRNA pseudouridine synthase A, mitochondrial	XP_001658327.1
AAEL009062-PA	A19	Uncharacterized	Voltage-dependent calcium channel	XP_001659677.2
AAEL009533-PA	A68	F-box protein 25/32, Fox0 signaling pathway	F-box only protein 25 (*Aedes aegypti*)	XP_001660205.1
AAEL009824-PA	A20, A67	Ubiquitin specific protein 9/faf	Probable ubiquitin carboxyl-terminal hydrolase FAF (*Aedes aegypti*)	XP_021705402.1
AAEL010440-PA	A67	Bud22/Serum response factor-binding protein 1	Nucleolin (*Aedes aegypti*)	XP_001660823.1
AAEL010962-PA	A18, A19	No match	Gustatory receptor 73 (*Aedes aegypti*)	NP_001345229.1
AAEL012615-PA	A67	No match	Uncharacterized protein LOC5576554 (*Aedes aegypti*)	XP_021705756.1

^1^ Putative function according to VectorBase: *Aedes aegypti* protein database: http://www.vectorbase.org/downloads. ^2^ Blastp-NCBI using non-redundant protein sequence (nr) and *Aedes aegypti* (taxid: 7159) databases at: https://blast.ncbi.nlm.nih.gov/Blast.cgi.

**Table 2:** Proteins found in extracellular vesicles from DENV-infected mosquito cells.

Gene ID VectorBase	Protein Vector Base	Putative Function-Vector Base ^1^	Putative Function-Blastp ^2^	Accession
AAEL000511	AAEL000511-PC	Acetylcholinesterase (Fragment)	Acetylcholinesterase isoform X1 (*Aedes aegypti*)	XP_001656977.3
AAEL001493	AAEL001493-PC	Laminin, N-terminal	Laminin subunit alpha-1 isoform X1 (*Aedes aegypti*)	XP_021700673.1
AAEL002675	AAEL002675-PA	Arginase	Arginase, hepatic (*Aedes aegypti*)	XP_001662057.1
AAEL003402	AAEL003402-PB	Sphingomyelin phosphodiesterase	AAEL003402-PB (*Aedes aegypti*)	EAT45277.1
AAEL003413	AAEL003413-PA	F-spondin	Spondin-1 (*Aedes aegypti*)	XP_001656777.2
AAEL003723	AAEL003723-PA	C-Type Lysozyme (Lys-A)	Lysozyme-like (*Aedes aegypti*)	XP_021699294.1
AAEL005951	AAEL005951-PC	Lipid storage droplets surface binding protein	Lipid storage droplets surface-binding protein 1 isoform X1 (*Aedes aegypti*)	XP_021693333
AAEL006240	AAEL006240-PA	purple acid phosphatase, putative	Select seq ref|XP_001651840.1|	XP_001651840.1
AAEL006434	AAEL006434-PA	Serine protease, putative	Serine protease 7 isoform X2 (*Aedes aegypti*)	XP_021703558.1
AAEL007992	AAEL007992-PB.	Trypsin, putative	Serine protease 7 isoform X1 (*Aedes aegypti*)	XP_021693694.1
AAEL009038	AAEL009038-PB	Prolylcarboxypeptidase, putative	Putative serine protease F56F10.1 (*Aedes aegypti*)	XP_021697410.1
AAEL009345	AAEL009345-PA	Prohibitin	Protein 1(2)37Cc (*Aedes aegypti*)	XP_001653792.1
AAEL011271	AAEL011271-PA	PDCD6IP	AAEL011271-PA (*Aedes aegypti*)	EAT36654
AAEL012326	AAEL012326-PA	Calmodulin family	AAEL012326-PA (*Aedes aegypti*)	EAT35514.1
AAEL013620	AAEL013620-PA	Ras-related protein	AAEL013620-PA (*Aedes aegypti*)	EAT34116.1
AAEL013952	AAEL013952-PE	Prohibitin	AAEL013952-PA (*Aedes aegypti*)	EAT33777.1
AAEL014566	AAEL014566-PD	Wingless	AAEL014566-PA, partial (*Aedes aegypti*)	EAT32499.1
AAEL015038	AAEL015038-PA	Palmitoyl-protein thioesterase	Palmitoyl-protein thioesterase 1 (*Aedes aegypti*)	XP_001650360.2
AAEL015235	AAEL015235-PA	Flotillin subfamily	AAEL015235-PA, partial (*Aedes aegypti*)	EAT32605.1
AAEL017301	AAEL017301-PA	Elongation factor 1-alpha	AAEL017301-PA (*Aedes aegypti*)	EJY57625.1
AAEL017982	AAEL023321-PA	HSP70	Heat shock 70 Cb (*Aedes aegypti*)	ACJ64198.1
AAEL020330	AAEL020330-PA	Unknown	Heat shock protein 70 A1-like (*Aedes aegypti*)	XP_021693654.1
AAEL021904	AAEL021904-PA	Unknown	Sushi, von Willebrand factor type A, EGF and pentraxin domain-containing protein 1 (*Aedes aegypti*)	XP_021702720.1
AAEL024406	AAEL024406-PB	Unknown	Uncharacterized protein LOC5572108 isoform X3 (*Aedes aegypti*)	XP_021699023.1

^1^ Putative function according to VectorBase: *Aedes aegypti* protein database: http://www.vectorbase.org/downloads. ^2^ Blastp-NCBI using non-redundant protein sequence (nr) and *Aedes aegypti* (taxid: 7159) databases at: https://blast.ncbi.nlm.nih.gov/Blast.cgi.

## Abbreviations

qRT-PCR	Quantitative Reverse Transcription PCR
EV	Extracellular Vesicle
